# Antigenic cartography of H1N1 influenza viruses using sequence-based antigenic distance calculation

**DOI:** 10.1186/s12859-018-2042-4

**Published:** 2018-02-12

**Authors:** Christopher S. Anderson, Patrick R. McCall, Harry A. Stern, Hongmei Yang, David J. Topham

**Affiliations:** 1 New York Influenza Center of Excellence at David Smith Center for Immunology and Vaccine Biology, Department of Microbiology and Immunology, University of Rochester School of Medicine and Dentistry, Rochester, NY USA; 2 Center for Integrated Research Computing, University of Rochester, Rochester, NY USA; 3 Department of Biostatistics and Computational Biology, University of Rochester School of Medicine and Dentistry, Rochester, NY USA

**Keywords:** Antigenic cartography, Antigenic distance, H1N1, Influenza, Hamming distance, Hemagglutinin

## Abstract

**Background:**

The ease at which influenza virus sequence data can be used to estimate antigenic relationships between strains and the existence of databases containing sequence data for hundreds of thousands influenza strains make sequence-based antigenic distance estimates an attractive approach to researchers. Antigenic mismatch between circulating strains and vaccine strains results in significantly decreased vaccine effectiveness. Furthermore, antigenic relatedness between the vaccine strain and the strains an individual was originally primed with can affect the cross-reactivity of the antibody response. Thus, understanding the antigenic relationships between influenza viruses that have circulated is important to both vaccinologists and immunologists.

**Results:**

Here we develop a method of mapping antigenic relationships between influenza virus stains using a sequence-based antigenic distance approach (SBM). We used a modified version of the *p-all-epitope* sequence-based antigenic distance calculation, which determines the antigenic relatedness between strains using influenza hemagglutinin (HA) genetic coding sequence data and provide experimental validation of the *p-all-epitope* calculation. We calculated the antigenic distance between 4838 H1N1 viruses isolated from infected humans between 1918 and 2016. We demonstrate, for the first time, that sequence-based antigenic distances of H1N1 Influenza viruses can be accurately represented in 2-dimenstional antigenic cartography using classic multidimensional scaling. Additionally, the model correctly predicted decreases in cross-reactive antibody levels with 87% accuracy and was highly reproducible with even when small numbers of sequences were used.

**Conclusion:**

This work provides a highly accurate and precise bioinformatics tool that can be used to assess immune risk as well as design optimized vaccination strategies. SBM accurately estimated the antigenic relationship between strains using HA sequence data. Antigenic maps of H1N1 virus strains reveal that strains cluster antigenically similar to what has been reported for H3N2 viruses. Furthermore, we demonstrated that genetic variation differs across antigenic sites and discuss the implications.

**Electronic supplementary material:**

The online version of this article (10.1186/s12859-018-2042-4) contains supplementary material, which is available to authorized users.

## Background

The Influenza A Virus (IAV) causes hundreds of thousands of hospitalizations and tens of thousands of deaths each year [1]. On average, about 5%–20% of the population will be infected each year [2]. The mutation rate of the virus genome is estimated to be 2.3 × 10^− 5^ mutations per nucleotide per infected-cell [3], with thousands of cells infected during the course of the disease [4]. This high mutability provides an evolutionary landscape that allows quick adaption to its ever-changing environment (i.e. population immunity). By mutating the proteins that cover the viral coat, the virus can escape antibody-mediated neutralization that occurs from antibodies binding to the surface of the virus. The goal of vaccination efforts is to elicit antibodies towards these neutralizing regions before exposure to the virus occurs, therefore providing protection [5]. Annual reformulation of the influenza vaccine is an attempt to keep population immunity up-to-date against these ever-changing viruses.

In order to keep a population immune to emerging strains, viruses are continuously sampled from the population. Virus strains are selected for use in the annual vaccine using both genetic and antigenic data [6]. The genetic data is quickly available publically and spans over a hundred years with data from tens to hundreds of viruses each year. This rich data set allows a unique opportunity to explore antigenic relatedness of the viruses that have circulated in the human population since the emergence of the 1918 H1N1 pandemic and whose descendants have almost continuously circulated in humans over the last century.

Although antigenic distances between vaccine strains have been measured experimentally [7–9], these studies use only a small fraction of the viruses that have been isolated and do not include the many genetically unique strains that circulate annually. Typically, measurement of the antigenic relatedness between viruses involves production of convalescent ferret antiserum and use of functional antibody binding assays, such as the hemagglutination inhibition (HAI) assay. Although these techniques are still the gold standard for vaccine choice, the cost and time intensive nature make these assays impractical. In many cases, ferret approaches are prohibitively expensive and not practical as an antigenicity model for the vast majority of laboratories worldwide. Graphical representation of antigenic distances are typically done using a dimension reduction approach known as antigenic cartography [8]. Antigenic cartography results in a two dimensional “map” in which distances between viruses on the map represent the antigenic distances between strains. These maps allow intuitive understanding of the antigenic relationships between large sets of viruses.

Few studies have provided comprehensive comparisons of the antigenic relationships between H1N1 HA proteins (including both pre- and post-2009 pandemic H1N1 strains). In recent years, HAI assay data produced annually by global health organizations (used to estimate antigenic distance between HA proteins) has become publically available, but these assays mostly cover recent isolates, and therefore antigenic measurements of older strains is limited. Recently, Liu et al. 2015 created a method to predict H1N1 antigenic clusters using a machine learning approach [10], this method can predict HAI based antigenic clusters, but its usability to predict non-HAI assay data is not known. Furthermore, the Liu et al. method provides only qualitative information about antigenic relationships and does not provide quantitative antigenic distance measurements. Fortunately, many studies have shown that antigenic distances can be estimated using genetic sequence data of the HA protein alone, but extensive validation of these methods are lacking [11–22].

Some sequence-based antigenic distance estimates have indeed been validated both by experimentally derived antigenic distance measurements of influenza virus strains [13, 19, 23], as well as by accurately predicting vaccine efficacy [11, 14, 22]. Other methods have been developed that merge sequence information and immunological assay data [7, 9, 12, 24, 25], but these approaches are limited to situations in which immunological assay data is available. A comparison of sequence-based antigenic distance measurement approaches demonstrated that those that focused on HA antigenic sites are most correlated with ferret antiserum-based antigenicity measurements [13]. Furthermore, most work regarding sequence-based antigenic distance estimates has focused on H3N2 strains and the correlation of antigenic distance estimates and immunological measurements has not been determined.

Sequence variation maps similar to antigenic cartography have been created previously [17, 21, 26], but antigenic maps of H1N1 viruses based on sequence-based antigenic distance calculations have not been created. Antigenic maps are based on pivotal work by Lapedes et al [27], Smith et al. [8], and are based on theoretical work by Perelson et al. [28]. Once (antigenic) distances are determined, many methods exist that can be used to reduced the dimensionality of the data and construct a map [29], allowing easy visualization and intuitive understanding of the antigenic relationships between viruses [8]. Here we use classical (metric) multidimensional scaling developed by Gower [30] to create an antigenic map of H1N1 viruses. We calculated and mapped sequence-based antigenic distance estimates between thousands of genetically unique H1N1 viruses that have circulated since the 1918 H1N1 pandemic. We compared our results to immunological measurements of relatedness using traditional methods.

## Methods

### HA protein sequence acquisition

HA protein sequences were obtained using the Influenza Resource Database [31]. Protein sequences were filtered using the following criteria: Subtype: H1N1; Protein: HA; and Host: human. Quality control was performed on the sequences by removing sequences containing missing or aberrant amino acids (i.e. “x”, “-”). Sequences not containing the start (M) or terminal amino acids (CI) and not containing the full coding sequence (565, 566 amino acids) were also removed. The first instance (by submission date) was used when identical protein sequences were found. Filtering resulted in 4838 unique HA sequences with lengths of 565 or 566 amino acids. Sequences were then aligned using the muscle algorithm [32].

### Antigenic distance estimation

An information theory based approach, *p-all-epitope*, developed by Deem et al. [11, 15] was used to calculated antigenic distances. Additionally, these distances were scaled to reflect a 20-dimensional immunological shape space described by Smith [33]. In order to calculate antigenic distances between HA proteins, protein sequences were aligned using the muscle algorithm [32]. This approach considers aligned amino acid sequences as character strings and computes the numbers of positions that do not match. The resulting value is known as the Hamming distance [34] between the amino acid sequences. For H1N1, these mismatches are only counted if they occur in five canonical H1N1 HA antigenic sites (Sa, Sb, Ca1, Ca2, Cb) comprised of amino acid regions (H1N1 numbering): Sa: 141–142, 170–174, 176–181; Sb: 201–212; Ca1: 183–187, 220–222, 252–254; Ca2: 154–159, 238–239; Cb 87–92 [35], with numbering beginning with the start codon methionine after muscle alignment. Antigenic site-specific mismatches are then divided by the total number of amino acids in the antigenic site and these percentages are then multiplied by 20 leading to distances on a scale from 0 to 20 representing a 20 dimensional immunological shape space (equation 1). This value we will refer to as “epitopic” distance to distinguish it from antigenic distance, although in reality the antigenic site probably consists of multiple antibody binding sites. Antigenic distances were calculated by averaging the epitopic distances for each antigenic site. Hamming distance calculations were implemented in a C program that takes a FASTA file containing sequences as input, and outputs a matrix of Hamming distances. The program was parallelized using OpenMP and run on the BlueHive linux cluster maintained by the Health Sciences Center for Computational Innovation and Center of Integrated Research Computing at University of Rochester.

1 $$ {ED}_{i,y}=\frac{number\ of\ amino\ acid\ changes\ in\ antigenic\ site}{total\ number\ of\ amino\ acid s\ in\ antigenic\ site}\times 20 $$

2 $$ {AD}_{i,y}=\frac{ED_{Sa}+{ED}_{Sb}+{ED}_{Ca1}+{ED}_{Ca2}+{ED}_{Sb}\ }{5} $$

Where *i* an *y* represent HA sequences from influenza virus strains and ED_xx_ represent antigenic sites: Sa, Sb, Ca1, Ca2, and Sb.

### Dimension reduction

The antigenic distances between proteins are structured into an *n x n* square-distance matrix (see Additional file 1). Given that each HA protein is described by antigenic distances to 4838 HA proteins, the data must be reduced in order for it to be graphed. Classic (metric) multidimensional scaling (MDS) can be used to preserve the distances between a set of observations in a way that allows the distances to be represented in a two dimensional space. This two dimensional space is similar to a topographical map, where the distances on the map between two HA proteins can be applied to a scale in order to obtain the antigenic distance. These maps are useful when trying to understand the antigenic relationships between a large set of HA proteins. In this way, each HA protein can be described using only a few values, allowing the data to be graphed. MDS was performed as previously described by Gower [30]. In short, MDS first constructs an n-dimensional space using the distance matrix in which all distances are conserved and Euclidian and then principal component analysis is performed. Goodness-of-fit (GOF) calculations were performed as previously described [36]. MDS and GOF were carried out using the *cmdscale* package in R. Color for each point in the antigenic maps was determined using hierarchical clustering of the antigenic distances used for Fig. 3. Hierarchical clustering was performed using the *hclust* R-base function. The *cutree* R-base function was used to subset hierarchical clustering into groups and the number of groups was determined empirically with 8 groups (k = 8) chosen. Vaccine/historical strains were labeled on the map as such: A/Brisbane/59/2007 (BR07), A/Solomon Islands/3/2006 (SI06), A/New Caledonia/20/1999 (NC99), A/Singapore/6/1986 (SI86), A/Beijing/262/95 (BE95), A/Taiwan/1/86 (TA86), A/Chile/1/83 (CH83), A/USSR/90/77 (US77), A/Fort Monmouth/1/1947 (FM47), A/Denver/1/1957 (Denv57), A/Marton/43 (MA43), A/Puerto Rico/8/34 (PR34), A/NWS/33 (WS33), A/South Carolina/1/1918 (SC18), A/New Jersey/76 (NJ76), A/California/04/2009 (CA09).

### Experimental validation

Mouse monoclonal antibodies were obtained from Influenza Reagent Resource (Cat#:FR-503, FR-495, FR-505) and BEI Resources (Cat# NR-13452). Ferret antiserum was obtained from Influenza Reagent Resource (Cat# FR-359, FR-388, FR-952, FR-953, FR-954, FR-955). Recombinant HA proteins were obtained from Influenza Reagent Resource (Cat#: FR-67, FR-692, FR-65, FR-180, FR-699) and BEI Resources (Cat# NR-19240, NR-48873). Chimera proteins were a gift from Dr. Florian Krammer from Mount Sinai (NY).

Recombinant HA proteins were coated on MaxiSorb 96-well plates (ThermoSci; 439,454) overnight at 4 °C. Plates were blocked with 3% bovine serum albumin (BSA) in phosphate buffered saline (PBS) for 1 h at room temperature. Ferret serum was diluted 1:1000 in PBS/0.5% BSA/0.05% Tween-20. Monoclonal antibodies were diluted to a concentration 15 μg/well. Diluted ferret serum or monoclonal antibodies were incubated overnight at 4 °C. Plates were washed and incubated with alkaline phosphatase (AP)-conjugated secondary antibody (Southern Biotech 1030–04 or LSBio LS-C61241) for 2 h at room temperature. Plates were washed and developed using AP substrate (ThermoSci 34,064). Mouse monoclonal antibody titers for each HA were derived from a standard curve created using C179 universally HA binding monoclonal antibody (Takara).

HAI data was curated from the WHO Collaborating Centre for Reference and Research on Influenza National Institute for Medical Crick Institute data repository and traditional antigenic distances were calculated [8]. To account for the abundance in coverage of new viruses and lack of HAI data on older strains, HAI data was only included where both recent and older stains were used in the assay.

The SBM method was validated by correlation analysis. The association between antigenic distance measurements and antibody titers were explored by Linear regression, and confirmed by Spearman correlation analysis. *P*-values were determined using either linear regression (*lm* stats package, R) or t-test (*t.test* stats package, R). P-values less than 0.05 were considered significant. Spearman correlations were performed using the base stats package in R. Further, ROC analysis was applied to test the performance of the SBM method. By validating the SBM against similarity mapping based on antibody titers, we assessed its sensitivity, specificity and the corresponding ROC curve. The RORC package in R was used for the analysis.

### Reproducibility

Reproducibility was determined by randomly sampling sequences from the 4838 sequences used to create the antigenic map in Fig. 3. This subset of sequences was then used to create an antigenic map and the distances between all points were calculated. The distances between all points were summed and compared to the summed distances of the same strains in the original 4838 sequence map. Fifty samplings were taken for each condition and precision was reported as the mean percentage. Reproducibility error for each condition was estimated by calculating the standard deviation between samplings.

Sensitivity was determined throμgh Receiver operating characteristic (ROC) analysis for each antibody titer measurement. Antibody binding data was converted into binary variables by calculating the relative difference in antibody titer or binding value (μg/ml, absorbance, titer) between matched serum/antibody and virus/HA strains (homologous value) and non-matches (heterologous value). Values two-fold or less than the homogenous values were considered as similar (value = 0) and those greater than 2-fold lower were considered dissimilar (value = 1). For HAI data, an additional definition of similarity was used by defining serum titers greater than 1:40 (i.e. 1:80, 1:160, …) were considered similar (value = 0).

Antigenic distances were converted into binary values by choosing a cutoff where values below that cutoff are given a value of 1 and those above that cutoff are given a value of 0. Sensitivity was calculated by dividing the number of true positives by the total number of positives. Specificity was calculated by dividing the number of true negatives by the by the total number of negatives. Overall accuracy at each cutoff was determined by dividing the sum of the number of true positives and true negatives by the sum of the number positive samples and negative samples.

## Results

### Antigenic distance estimates

The antigenic distance (AD) between HA protein antigens of H1N1 viruses were calculated for 4838 HA protein sequences (Additional file 1). The maximum antigenic distance between the strains was 33 with a mean antigenic distance between all strains of 10.4. Antigenic distances were bimodally distributed with few comparisons having antigenic distances between 4 and 8 and all antigens had similar mean values with similar distributions (Additional file 2: Figure S1).

Antigenic distances between vaccine strains ranged from 0 to 29 (Additional file 3: Table S1) with an average of 15.18 AD. Antigenic distance between strains generally correlated well with differences in the year of isolation between strains with the exception of the 2009 virus (CA09), which had a long antigenic distance (26) to BR07 and a shorter antigenic distance (10) to the 1918 virus (SC18), suggesting distal ancestry. Moreover, early twentieth century viruses generally had a lower AD to CA09 compared to late twentieth century and early twenty-first century strains.

### In vitro validation of antigenic distances

Since antigenicity differences are defined by differences in antibody binding, antigenic distances can be used to predict antibody binding between two antigens. To this end, data from three independent antibody binding assays were analyzed to assess the ability of sequence-based antigenic distances to predict antibody cross-reactivity. The standard antigenicity model used by the CDC and WHO is to infect an animal model (typically ferret) with influenza virus strain “X” and measure the resulting antiserum reactivity towards strain “Y” using the functional antibody-binding assay HAI. The assay measures the minimal antiserum titer needed to disrupt binding of the virus to sialic acid on red blood cells. In addition, antibody binding data to recombinant HA was obtained by enzyme-linked immunosorbent assay (ELISA) using both mouse monoclonal antibodies specific for historical strains, as well as anti-influenza ferret antiserum. Linear regression and both Pearson and Spearman correlations were used to assess the relationship between sequence-based AD and antibody binding. Spearman correlation is similar in nature to Pearson correlation except is non-parametric in that observations are ranked and correlations are determined based on those ranks. In this way, Pearson correlation determines the linear correlation between observations, while Spearman correlation determines the monotonic relationship between observations.

**Fig. 1:**
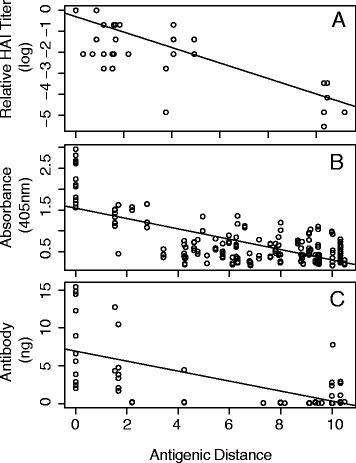
Comparison of Experimental Data with Sequence-based Antigenic Distance. **a** Relative HAI log titer of ferret antiserum. **b** ELISA absorbances of ferret antiserum to rHA. **c** Amount of mouse derived monoclonal antibodies bound to rHA by ELISA

HAI data was curated from the WHO Collaborating Center Crick Institute data repository. Data from 80 HAI assays were used for comparison and traditional antigenic distances were calculated. The HAI assay data includes virus strains and antiserum from viruses that span from 1977 to 2009 including 2009 pandemic-like strains with serum-strain/virus-strain antigenic distances ranging from 0 to 26. HAI derived antigenic distance significantly decreased linearly as sequence-based antigenic distance increased (Fig. 1a) and HAI derived antigenic distance and sequence-based antigenic distance was highly correlated (Pearson CC = − 0.66 *p* < 0.0001; Spearman CC = − 0.55 *p* < 0.0001).

Although HAI based antigenic distance estimates are the standard, recent reports have demonstrated that these assays are affected by other characteristics besides antigenicity (e.g. RBC affinity, NA binding, etc). Additionally, the HAI assays do not include virus strains that circulated prior to 1977. Therefore, to further validate the sequence-based antigenic distance calculations, we measured antibody binding using ELISA for strains that circulated from 1918 to 2009, including 1947 strains and 1977 strains covering most clusters and spanning the map with serum-strain/HA-strain antigenic distances ranging from 0 to 27. Plates were coated with recombinant HA (rHA) proteins and antibody binding of ferret antiserum to each rHA was determined. In these assays, rHA from each strain is bound to a plastic and therefore not affected by Sialic acid affinity of the virus or NA binding. Antibody titers significantly decreased with sequence-based antigenic distance measurements (Fig. 1b; Pearson CC = − 0.8 *p* < 0.0001, Spearman CC = − 0.79 p < 0.0001).

Although the HAI and ELISA assay binding data indicate that the sequence based method is valid, the lack of matching ferret antiserum-rHA (as is done with the HAI based method) does not allow us to account for differences in immune responses across the ferrets. Therefore, we used monoclonal antibodies derived from B cells of mice infected with various influenza strains isolated from 1918 to 2009 including 2009-pandemic like strains, 1947 and 1977-like strains with serum-strain/virus-strain antigenic distances ranging from 0 to 27. Standard curves were created for each rHA to allow better quantification of the amount of mAb bound to each rHA. In agreement with the HAI and ferret-ELISA assays, as sequence-based antigenic distance between the rHA and the infecting strain HA increased, the amount of antibody binding significantly decreased (Fig. 1c; Pearson CC = −.67 *p* < 0.0001, Spearman CC = − 0.41 *p* < 0.0001). Taken together, these results indicate that sequence-based antigenic distance calculations correlate well with immunological assay measurements of antigenicity, regardless of assay, and therefore can be used to estimate antibody cross-reactivity.

### H1N1 antigenic cartography

Distances between a set of observations, such as antigenic distances between HA antigens, can be visualized on a 2-dimensional graph using approaches known collectively as dimension reduction. For distance matrixes (i.e. dissimilarity matrix), classic MDS, also known as principal coordinate analysis, is appropriate. MDS projects the distances into a Euclidean space in a lower number of dimensions in a way that preserves the original distances [8]. Therefore, MDS was performed on the sequence-based antigenic distance matrix in order to create an antigenic map.

**Fig. 2:**
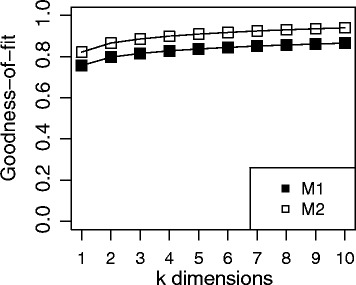
GOF After Dimension Reduction. Goodness-of-fit calculations after dimension reduction into k dimensions (1–10). Two methods were used to calculate the GOF depicted by open or closed boxes

Not all distance matrixes can be represented in 2-dimensions without significant loss of the distance data. Therefore, the preservation of the antigenic distances after MDS was determined. The R package for classical multidimensional scaling (cmdscale) returns two GOF statistics. One is the sum of the eigenvalues for the components S divided by the sum of the absolute value of all eigenvalues (Fig. 2, M1). The other is S divided by the sum of all positive eigenvalues (Fig. 2, M2) [37]. Plotting the GOF statistics as a function of k (the number of dimensions after MDS), it is possible to determine the number of dimensions that are necessary to adequately represent the data. In this way, a k is chosen when adding more dimensions do not significantly improve the goodness-of-fit. The GOF for a range of k values (k = 1–6) was determined (Fig. 2). The greatest increase in GOF was seen when k increased from 1 to 2 and only increased slightly thereafter. Two dimension (k-2) reduction lead to GOFs of 0.80 and 0.87 for the two methods used. Increasing k to 3 or 4 only slightly increased the GOF (0.82 & 0.89; 0.83 &0.90, respectively). Therefore, multidimensional scaling using k = 2 was chosen for creating the antigenic map.

**Fig. 3:**
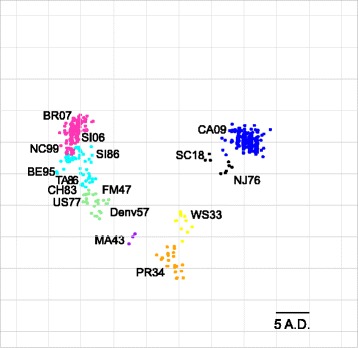
Sequence-based Map of H1N1 HA Proteins. Antigenic cartography of sequence-based antigenic distance calculations for HA protein sequences on 4838 H1N1 viruses. Each point on the map represents a HA protein antigen. The distance between two HA protein antigens on the map represents the antigenic distance between the two antigens. Historical Strains are labeled. Points are colored based on categorical hierarchical clustering

We constructed an antigenic map using the sequence-based antigenic distance calculations for the 4838 viruses and applied multidimensional scaling (Fig. 3). Viruses generally cluster into distinct groups across the map. There was a clear relationship with time from FM47 to BR07, demonstrating the continuous antigenic evolution of H1N1 viruses that has occurred during the last 50 years. Interestingly, SI06 and BR07 had a shorter antigenic distance (4 AD), but appeared to separate into small, but distinct, clusters, although this discrimination was not found using our hierarchical clustering-based method to coloring clusters. There was also a general separation between SC18-like, NJ76-like, and CA09-like virus strains and other viruses, consistent with phylogenetic analysis [38]. WS33 and PR34 strains separated from both SC18-like strains and FM47-like strains, which may indicate that antigenic changes have occurred as an artifact of being generated in the laboratory. Additionally, US77 and FM47 clustered together, consistent with the belief that the FM47 virus has remained unchanged for 30 years and reemerged unnaturally causing the 1977 pandemic. Taken together, H1N1 viruses cluster into groups on the map in a way that reflects what is known about the antigenic relationships between historic strains.

**Fig. 4:**
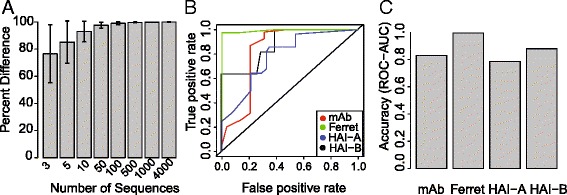
Precision and Accuracy. **a** Precision of the distances between antigens on the map was determined using random sampling of HA sequences (0–4000 sequences) to construct the map. Each sampling was performed 50 times. Precision is given as the mean percent difference between distances of randomly chosen sequences and distances in original map. Error bars represent the standard deviation between results of sequence samplings. **b** ROC-AUC measurements of accuracy for each of assay type and criteria, HAI-A and HAI B represent 2-fold and 1:40-dilution criteria, respectively. **c** ROC curves over the range of antigenic distances cutoffs

To evaluate the overall performance of the antigenic map, we first set out to determine how dependent the map was on sequence sampling. Given the large number of sequences used to create the map (4838) we determined how the number of sequences used in the creation of the map affects the antigenic distances on the map. To determine precision, maps were created for different number of randomly sampled and distances between the original map and the sequence sampling map were compared. Overall, the precision of the model decreased (error increasing) as the number of sequences used to make the map decreased. Distances were well conserved, over 90% agreement, when 10 or more sequences were used (Fig. 4a) and over 80% agreement with 5 sequences were sampled. Although the average agreement was over 75%, error was increased. Therefore, the model is highly reproducible when 10 or more sequences are used in the map creation.

Next we studied the ability of the antigenic distances predicting the similarity between strains determined by the ELISA/HAI antibody binding data. In this way, we can determine how accurate the map is at predicting cross-reactive antibody titers. Since experimental variations of HAI titers within a 2-fold range are considered similar [39], similarity of the antibody binding data (mAb-ELISA, ferret serum-ELISA, HAI data) was defined as titers/values 2-fold or less different than the matched serum/antigen value. ROC curves were produced using the range of antigenic distance cutoffs for the strains used in each assay. In this way, we can determine the ability of the model to predict decreases in cross-reactive antibody levels. Additionally, serum HAI titers of 1:40 have been shown to produce a 50% reduction in susceptibility to infection [39]. Therefore as an additional estimate of similarity, virus strains having a HAI titer of 1:40 or greater (e.g. 1:80, 1:160, …) were considered as similar to the serum strain.

The sensitivity (True positive rate) and specificity (False positive rate) of the model was determined over the entire range of antigenic distances using ROC curves (Fig. 4b). The ability of AD predicting similarity status was determined by calculating the area under the curve (AUC) from the ROC analysis. Area under the ROC curve can be used to evaluate the overall performance of the model. In general, areas between 0.5–0.7 are considered moderately useful, areas 0.7–0.9 as a good test, and greater than 0.9 as an excellent test [HajianTilaki:2013wh]. AUC varied by assay, and was greatest for the ELISA based assays (Fig. 4c). The AUC for the Ferret-ELISA data was 0.99 and mouse-ELISA was 0.82. For the HAI data, the 2-fold criteria had an AUC of 0.78 with the 1:40 titer having a 0.82 AUC. Taken together, the model is highly precise over a large range of input sequences and has a high degree of predictive accuracy for all three experimental measurements of virus antigenicity.

### Epitope-specific antigenic mapping

**Fig. 5:**
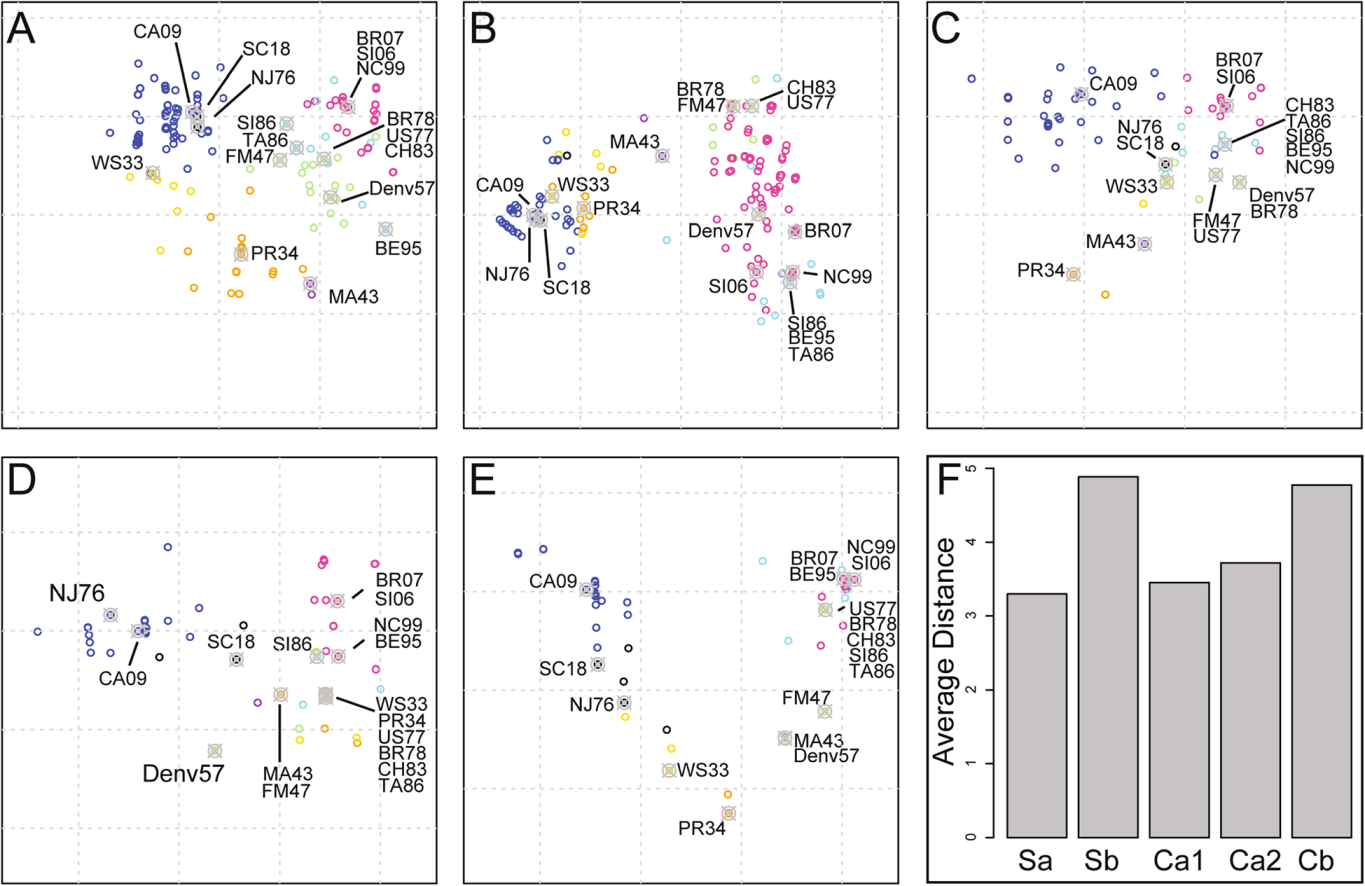
Epitopic Distances of H1 HA Epitopes. **a**-**e** Antigenic maps for each antigenic site (Sa, Sb, Ca1, Ca2, Cb, respectively). Strains were colored as in Fig. 2. **f** Average antigenic distances between all strains for each antigenic site

Given that HA regions evolve at different rates [40], we set out to establish if the pattern of antigenic relationships in the antigenic map between strains was similar for all epitopes. To do this, antigenic distance was calculated using only the amino acids of a single epitope for each map. This resulted in 5 antigenic maps representing the 5 HA antigenic sites: Sa, Sb, Ca1, Ca2, Cb (Fig. 5a-e). GOF was similar for all antigenic site maps (~ 0.8). Average antigenic distances for each epitope (epitopic distance) were similar, with Sb and Cb having the greatest antigenic variation (Fig. 3f). Overall, CA09 viruses clustered away from other strains with the exception of SC18 and NJ76, which were similar to the all-antigenic-site map (see Fig. 2). Interestingly, for the Ca1 antigenic site map CA09 did not cluster with SC18 or NJ76. Additionally, PR34 had a shorter antigenic distance to CA09 for the Sb antigenic site map, but not for the Sa map.

Strains isolated between 1947 through 2007 also generally clustered together. Although general trends are similar between antigenic site maps, many specific differences were found. For instance, NC99 and BR07 had similar distances in all antigenic site maps, while SI06 was similar in Sa, Ca1, Ca2, Cb, but not Sb. Additionally, CH83 and TA86 were similar in all epitopes except Sb. Interestingly, many strains had identical antigenic sites despite decades of separation between when they circulated. For example, WS33 had an identical Ca2 antigenic site as TA86 despite circulating over 50 years later. This may reflect uneven immune pressure against different epitopes. Taken together, antigenic site specific differences can be found between strains, although common patterns exist.

## Discussion

We present, for the first time, that an antigenic map of H1N1 proteins can be created using HA protein sequence data. We show that these sequence-based antigenic distance estimates correlate well with experimentally derived antigenicity measurements and demonstrate that antigenicity differs depending on antigenic site. Overall, our findings suggest that sequence-based antigenic distance measurements can be used as a surrogate for immunological based approaches and as input for antigenic cartography.

Previous work by Smith et al. [41] demonstrated that a 20 character Hamming distance space is best representative of H3N2 influenza HA antigens in the immunological shape space originally described by Perelson et al. [28]. This Hamming distance space is such that a 35% change in antigen sequence equals an antigenic distance of 7, the minimum distance between two HA proteins in which an antibody that recognizes one HA will not recognize the other. Therefore, in this 20-dimensional space, lower distances (between 0 and 7) indicate overlap of the recognizing antibodies, while larger distances (8–20) indicate no overlap. Our experimental in vitro results suggest that little binding occurs when distances are greater than eight, consistent with their findings.

Additionally, recent investigations have addressed the need to understand antigenic relationships between the viruses to which one is exposed early in life, as well as current vaccine strains, in order to predict immune responses [42, 43]. Consistent with these findings, we found that the 2009 pandemic strain resides closest to early twentieth century virus strains. The proximity of CA09 and SC18 viruses is consistent with reports by us [43] and others [44] showing that preexisting memory to head epitopes was responsible for increased immunity in individuals exposed to 1918 viruses. Furthermore, the close proximity of the 2009 pandemic strains and the New Jersey vaccine strain (NJ76) is consistent with increased immunity to the pandemic strain in NJ76 vaccinated individuals [45]. The large distance between the PR8 laboratory strain (PR34) and both CA09 and SC18 is likely due to accumulated mutations of this virus from repeated propagation in culture and is consistent with other reports that showed that low cross-reactivity occurs between these viruses in serum from infected animal models [46].

The distances between virus strains that circulated between 1977 and 2008 demonstrate continual antigenic drift of the virus over this period. This finding is consistent with reports for H3N2 viruses [8]. Additionally, previous work by Ren et al. used traditional antigenic cartography to map the antigenicity of H1N1 viruses that circulated from 1978 to 2008 [9]. Consistent with our findings, Ren et al. found similar continual drift between strains over the years. Furthermore, work by Bedford et al. integrated antigenic cartography and phylogenetic analysis for H1N1 viruses from 1977 to 2006 and demonstrated very similar clustering to that presented here, although separate clustering by US77-like strains and TA86-like strains was more pronounced using their method [7]. Taken together, our map agrees with other antigenicity studies of H1N1 viruses.

The comparison of antibody binding assay data and antigenic distance calculations demonstrates both the validity of our approach and the accuracy of the theoretical relationship between epitopes and paratopes in immunological shape space. It should be noted that clustering (gaps between groups of viruses) might occur due to lack of surveillance during a specific period. Clustering is undoubtedly occurring in this data. The lack of sequences between 1918 and 1933, despite documented circulation of the virus, clearly demonstrates these sampling gaps. Regardless, more recent strains also show clustering, demonstrating that sampling error is not the only cause of clustering in the data set. More studies are needed to address the cause of this clustering and distinguish clustering from sampling error.

Our method attempts to estimate shape differences between HA using changes in protein sequence instead of traditional HAI assays. HAI titers are a functional readout of the epitope/paratope interaction and are sensitive to experimental conditions. HAI measurements are affected by the affinity of the sialic-acid-binding-receptor on red blood cells, and differences in HAI titer may reflect these affinity changes [47]. Additionally, HAI titers are largely dependent on antibodies that bind near the sialic acid receptor-binding domain [48], and therefore these measurements are biased towards specific epitopes. Unlike these methods, the approach taken has not affected these experimental nuances. Therefore, our approach, or a similar approach, may lead to greater accuracy in predicting cross-reactive immunity, especially when differences in affinity to sialic acid exist among the strains.

It is important to acknowledge that the exact distances in the epitope-specific maps are sensitive to the amino acids chosen to represent the epitope. The specific epitope location on the HA protein, and therefore the amino acids making up that epitope, may differ depending on host species and genotype. Additionally, other studies have demonstrated that post-translational modification affects antigenicity [49], which is currently not captured in our method. Therefore, caution must be taken not to over interpret the findings presented here. Additionally, it has been demonstrated that changes in antigenic sites located close to the sialic acid binding domain of the H3N2 influenza viruses largely account for HAI assay differences [50, 51]. Therefore, future models may need to weight H1N1 antigenic sites in order to better predict HAI titers. Nonetheless, the fact that differences exist among epitopes is in line with experimental studies demonstrating that antibody mediated protection from virus is dependent on the epitope similarity of circulating strains and a strain in which the host was previously exposed [43, 44]. It is also important to note that it was not possible to experimentally validate individual epitopic distances. Future validation of epitopic distance should include more extensive monoclonal binding assays incorporating a panel of epitope specific monoclonal antibodies representative of the B cells initiated by infection or immunization. Taken together, these results present a need to better understand relationships between antigens at the epitope level. More estimates of the antigenic differences at the epitope level will improve our understanding of immunological shape space.

## Conclusion

SBM can be used to accurately estimate antigenic relationships across H1N1 influenza virus strains. H1N1 viruses form distinct antigenic clusters similar to what has been reported for H3N2 viruses. SBM correctly identified the large antigenic distances between the 2009 seasonal vaccine strain and 2009 pandemic virus strains as well as captured the short distance to the 1918 pandemic strains. Furthermore, we demonstrated that antigenic sites differ in their conservation. Altogether, SBM provides an alternative approach to traditional immune assays for antigenic distance estimates and can provide greater detail into the intra-antigenic relationships of the hemagglutinin protein.

## Additional files

Additional file 1: Antigenic distances for all strains used in the study. (CSV 53857 kb)

Additional file 2: Figure S1. Histogram of antigenic distance for all strains used in the study. (PDF 172 kb)

Additional file 3: Table S1. Antigenic distances for historical/vaccine strains. (CSV 749 bytes)

## Abbreviations

ELISA: Enzyme-linked immunosorbent assay
HA: Hemagglutinin
HAI: hemagglutination inhibition
IAV: Influenza A Virus
SBM: Sequence-based mapping
