# Supplementary figures and images for: Antigenic cartography of H1N1 influenza viruses using sequence-based antigenic distance calculation
Source: BMC Bioinformatics. 2018 Feb 12;19:51. doi: 10.1186/s12859-018-2042-4 (PMC5809904; doi:10.1186/s12859-018-2042-4)

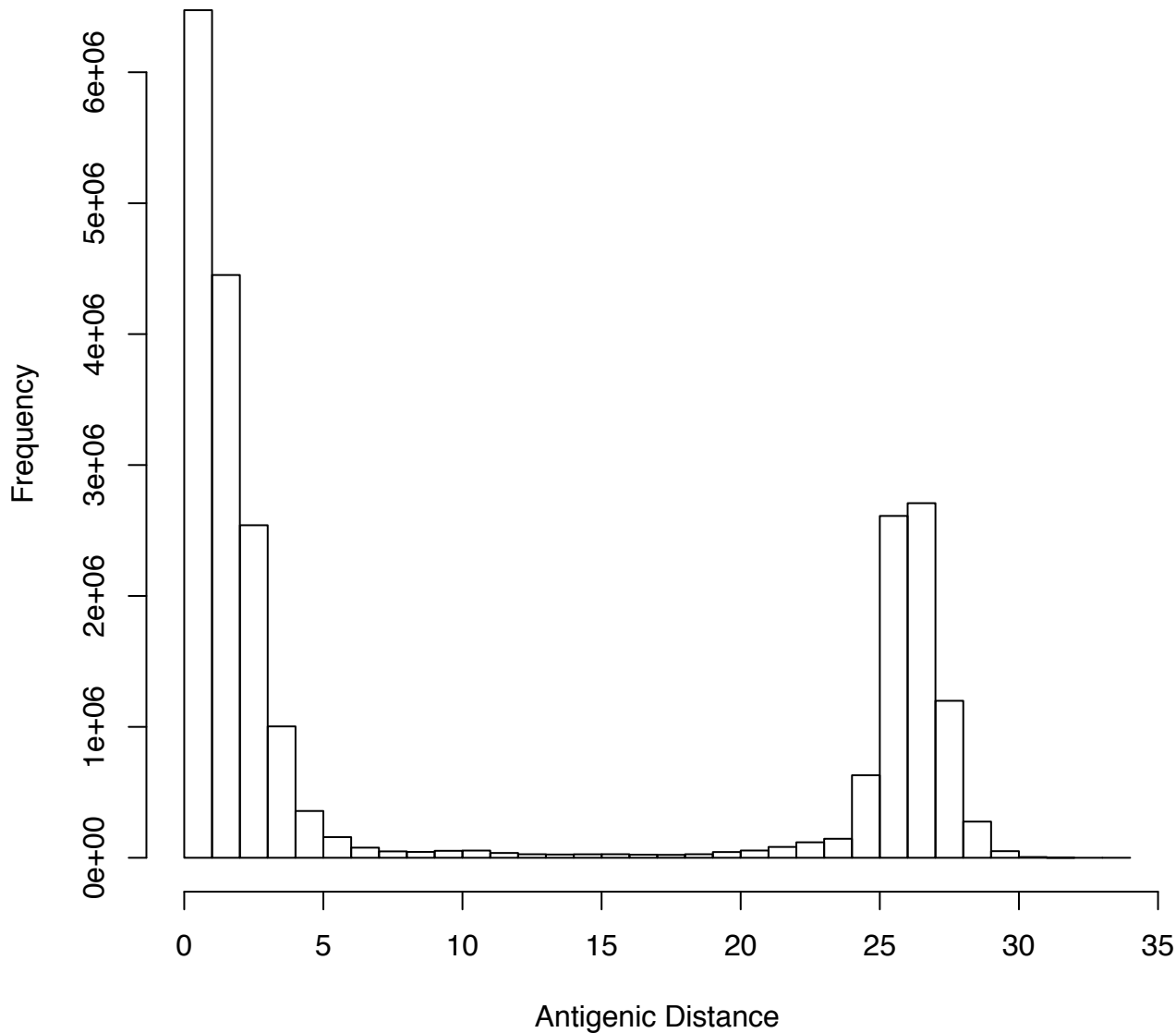

Figure S1: Histogram of Antigenic Distances  
Total of 23,406,244 antigenic distances for 4838 HA proteins.

Supplement: Supplementary file 2 — Histogram of antigenic distance for all strains used in the study. (PDF 172 kb) [file 12859_2018_2042_MOESM2_ESM.pdf]
